# Supplementary material for: The feather degradation mechanisms of a new Streptomyces sp. isolate SCUT-3
Source: Commun Biol. 2020 Apr 24;3:191. doi: 10.1038/s42003-020-0918-0 (PMC7181669; doi:10.1038/s42003-020-0918-0)
Supplement: Supplementary file 1 — Supplementary Information [file 42003_2020_918_MOESM1_ESM.pdf]

SUPPLEMENTARY INFORMATION

The feather degradation mechanisms of a new *Streptomyces* sp. isolate SCUT-3

Supplementary Figures 1–8

Supplementary Tables 1–5

Supplementary Figures

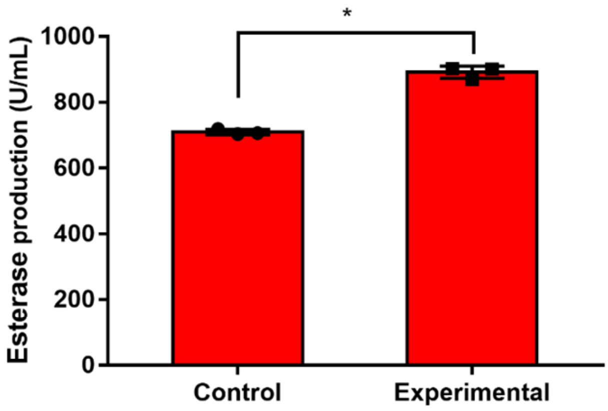

Supplementary Figure 1 | The effect of feather degradation products on microbial recombinant protein production; *n* = 3/group, **P*<0.05. The experimental group added 3% feather hydrolysate (from 10% SCUT-3 cultured CFM broth) to the control LB medium. *P*-values between groups were obtained by unpaired two-tailed Student’s *t*-test. All data were presented as mean ± SD.

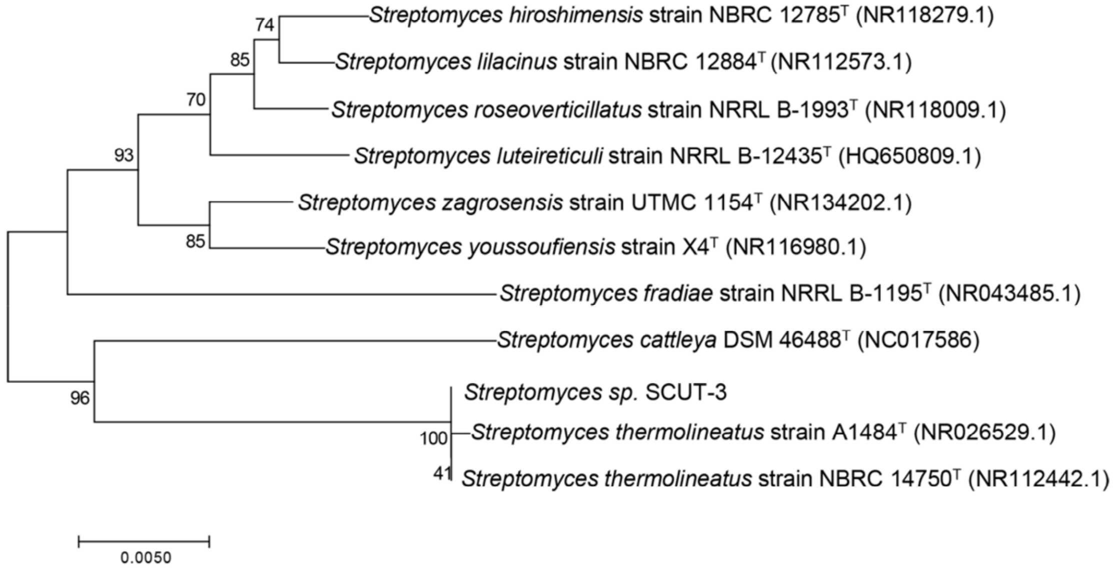

Supplementary Figure 2 | Phylogenetic tree constructed using the neighbor-joining method based on 16S rRNA gene sequences. The robustness of individual branches was estimated by bootstrapping with 1000 replicates. The bar represents an evolutionary distance of 0.005. Numbers at nodes indicate the bootstrap support (%). Accession numbers of 16S rRNA gene sequences included in the analysis are shown in brackets.

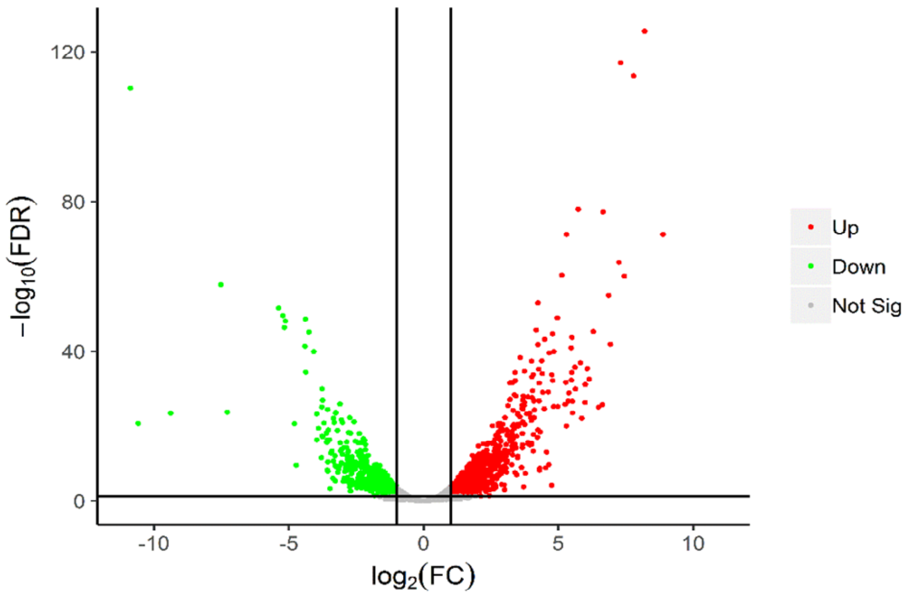

Supplementary Figure 3 | Volcano map illustrating transcriptome analysis of differentially expressed genes. Red dots, significantly up-regulated genes (log_2 FC > 1); green dots, significantly down-regulated genes (log_2FC < -1); gray dots, genes with no significant change in expression ($|log_2FC| < 1$).

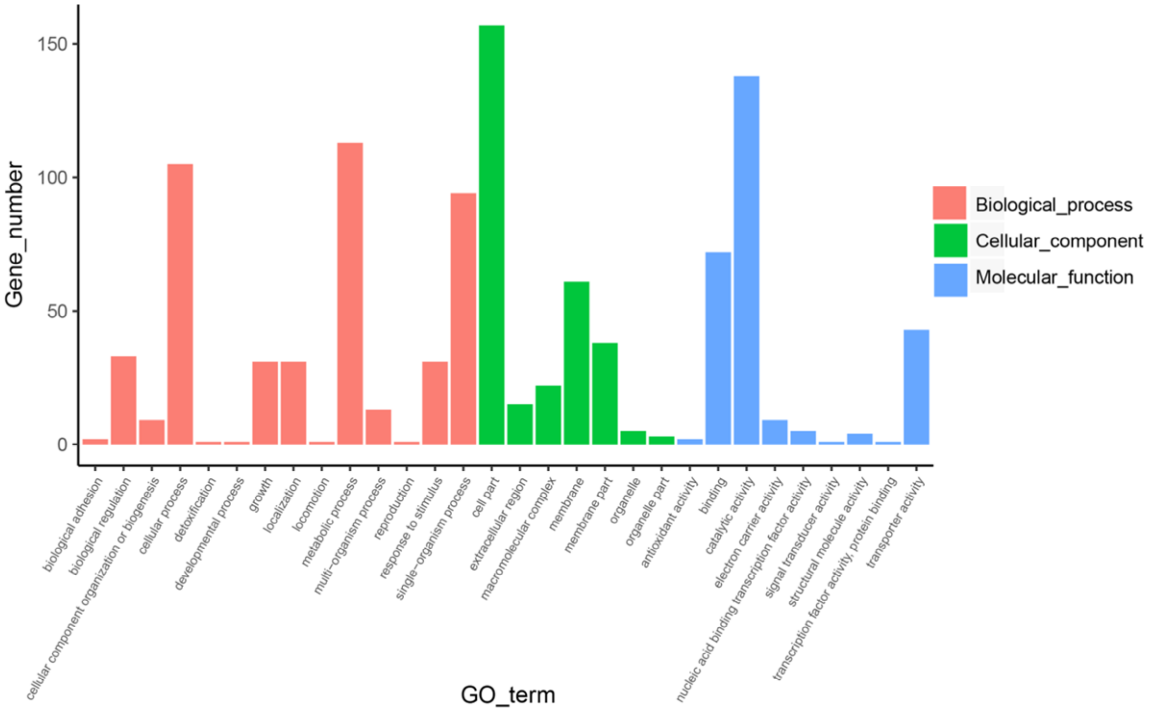

Supplementary Figure 4 | GO cluster analysis of DEGs. Red, green, and blue columns represent the number of DEGs associated with biological processes, cellular components, and molecular functions, respectively.

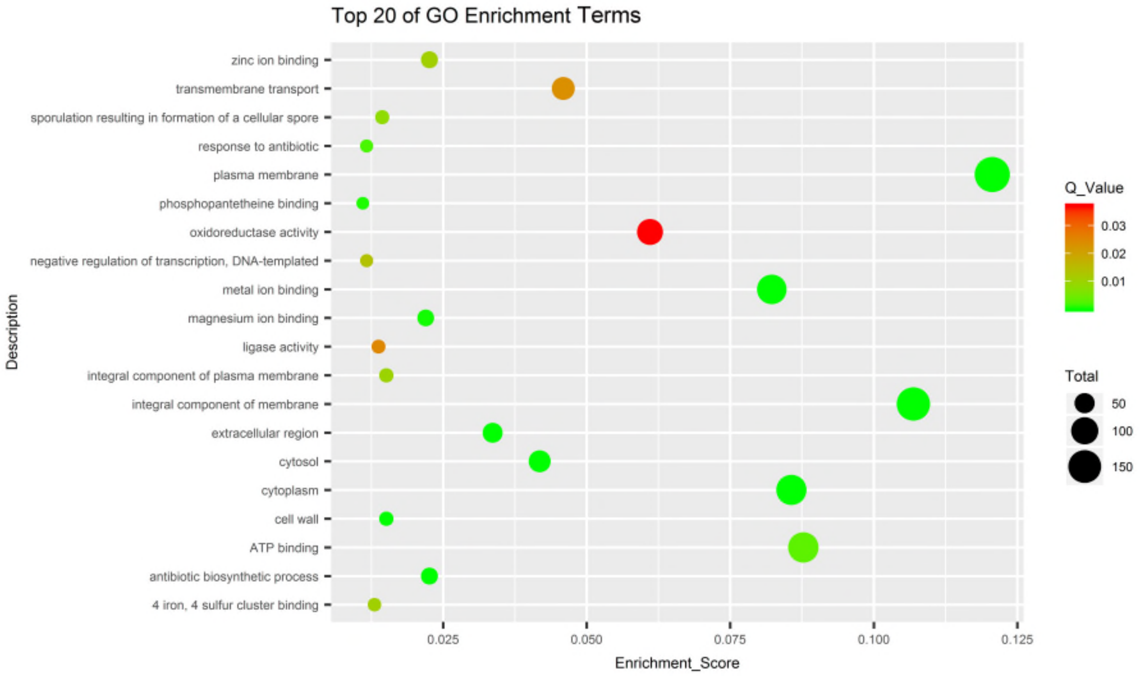

Supplementary Figure 5 | Top 20 GO enrichment terms among DEGs. Circle size indicates the number of DEGs in each entry, while color indicates q value.

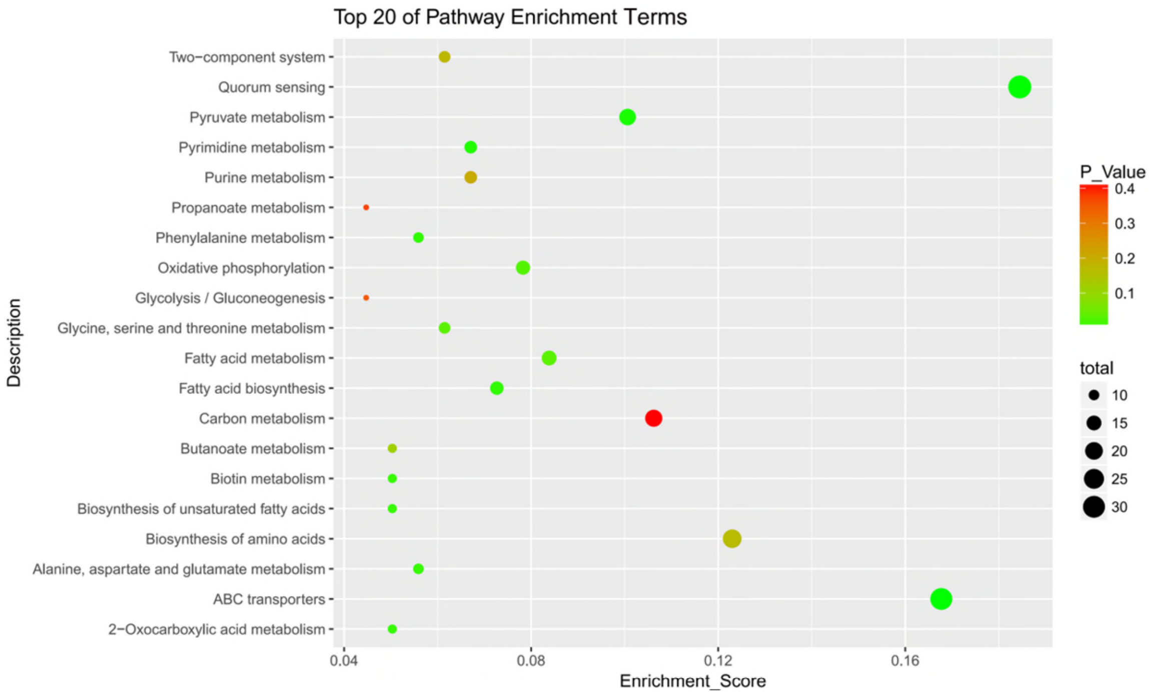

Supplementary Figure 6 | Top 20 KEGG enrichment terms among DEGs. Circle size indicates the number of DEGs, while the color indicates p value.

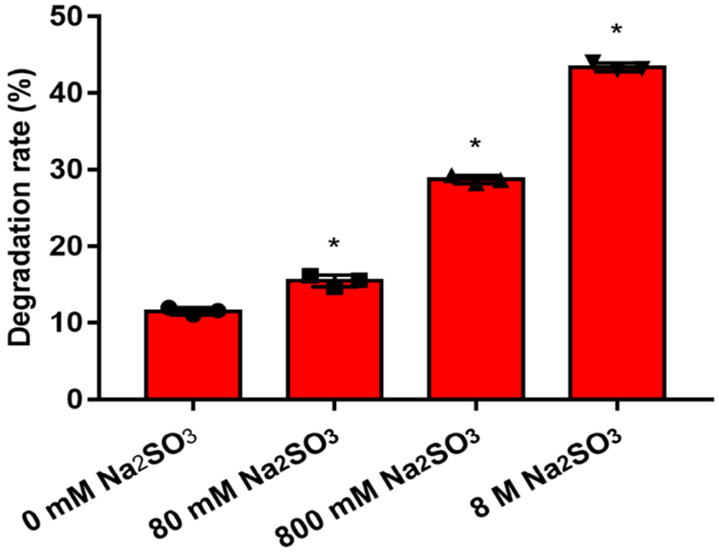

Supplementary Figure 7 | Effect of different concentrations of sulfite on feather degradation. *n* = 3/group, **P* < 0.05. *P*-values between groups were obtained by unpaired two-tailed Student's *t*-test. All data were presented as mean ± SD.

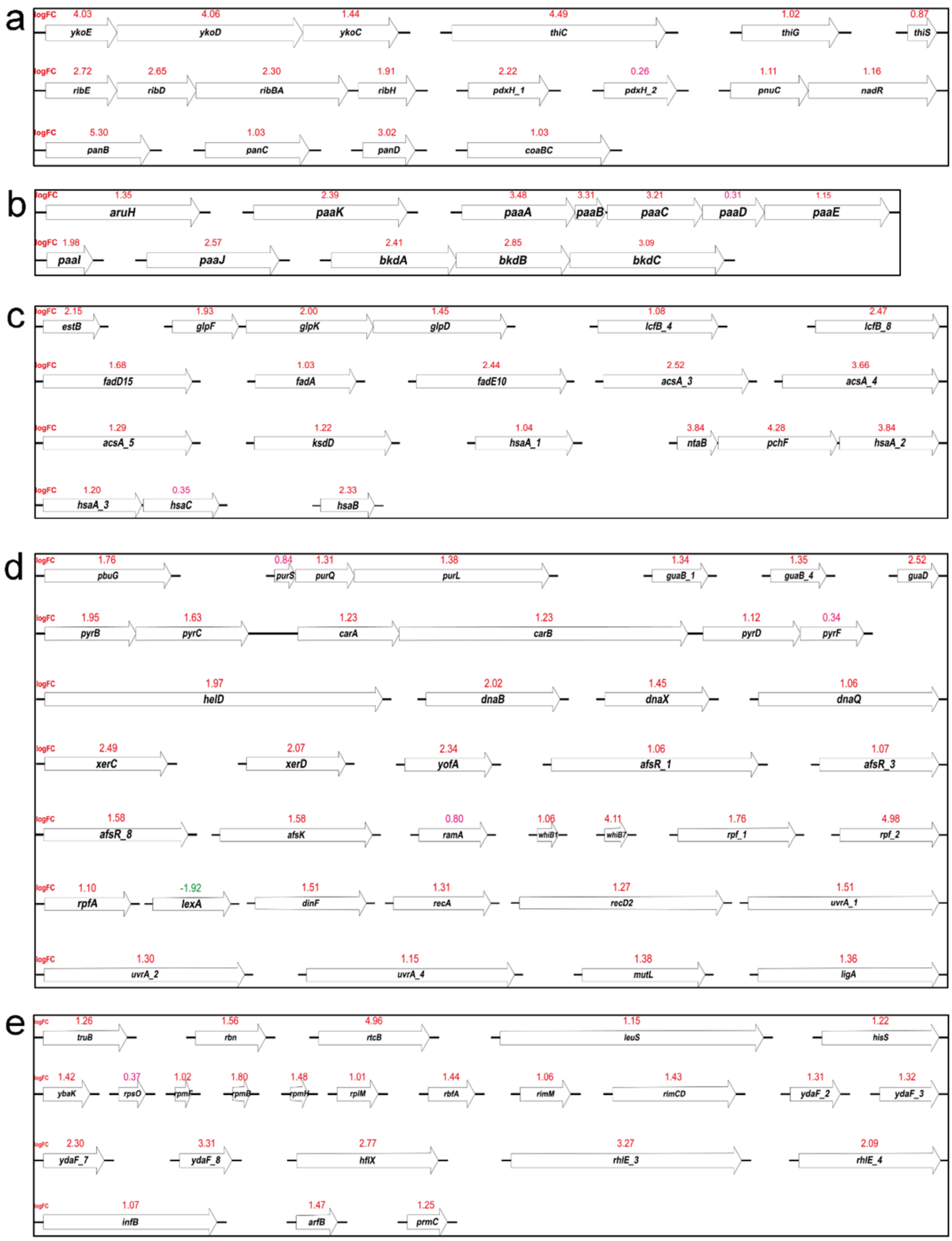

Supplementary Figure 8 | Significantly up-regulated genes involved in SCUT-3 feather utilization. Genes associated with **a** vitamin uptake and anabolism; **b** amino acid catabolism; **c** lipid catabolism; **d** DNA replication and cell division; and **e** transcription and translation. Numbers above genes indicate the log_2FC value for each gene (red > 1.0, 0 < pink < 1.0, green < -1.0).

Supplementary Tables

Supplementary Table 1 | Amino acid composition of untreated chicken feather, fermented feather meal, soluble free amino acids, and free amino acid/peptides in fermented feather.

Amino acid	Untreated chicken feather (%)	Fermented feather meal (%)	Free amino acids (%)	Free amino acids/peptides (%)
**Valine**	**7.86**	**7.72**	**14.56**	**8.10**
**Leucine**	**7.29**	**7.49**	**14.67**	**8.17**
**Isoleucine**	**4.56**	**4.49**	**7.75**	**4.72**
**Lysine**	**1.71**	**1.61**	**3.48**	**2.94**
**Methionine**	**0.46**	**1.38**	**-**	**1.63**
**Threonine**	**4.90**	**4.49**	**4.79**	**3.85**
**Phenylalanine**	**4.33**	**4.38**	**6.69**	**4.91**
**Tryptophan**	**-**	**-**	**1.35**	**0.72**
Histidine	0.46	0.23	0.38	0.69
Tyrosine	2.39	2.88	7.05	3.70
Serine	12.19	9.45	0.98	4.57
Glutamic acid	10.59	10.48	6.60	12.97
Arginine	7.06	5.99	4.48	3.86
**Cystine**	**8.20**	**5.88**	-	3.18
Aspartic acid	6.49	5.99	2.66	6.14
Proline	9.79	15.21	-	14.96
Alanine	4.33	5.07	13.82	7.64
Glycine	7.40	7.26	10.74	7.99
**Essential amino acids**	**31.11**	**31.56**	**49.00**	**35.73**

Supplementary Table 2 | Average genomic nucleotide identity analysis of SCUT-3 relative to 1222 other *Streptomyces* sp. strains (top 10 ANI strains are listed).

Species	Strain	Genome size (Mb)	ANI (%)	GenBank accession number
Streptomyces cattleya	DSM 46488	8.09	78.51	GCF_000237305.1
Streptomyces pathocidini	NRRL B-24287	5.17	78.42	GCF_001418495.1
Streptomyces sp.	ICBB 8177	6.32	78.37	GCF_003144095.1
Streptomyces cattleya	NRRL 8057	8.10	78.36	GCF_000240165.1
Streptomyces fradiae	NRRL B-1195	5.27	78.33	GCF_002154445.1
Streptomyces radiopugnans	CGMCC 4.3519	6.05	78.33	GCF_900110735.1
Streptomyces barkulensis	RC 1831	6.48	78.33	GCF_002843305.1
Streptomyces caatingaensis	CMAA 1322	7.05	78.29	GCF_001187435.1
Streptomyces alboverticillatus	NRRL B-24281	6.57	78.27	GCF_002150845.1
Streptomyces megasporus	NRRL B-16372	5.76	78.26	GCF_000718985.1

Supplementary Table 3 | Morphological and biochemical characteristics of SCUT-3 and *Streptomyces thermolineatus* A1484.

Characteristic	*Streptomyces* sp. SCUT-3	*Streptomyces thermolineatus* A1484
Culture characteristics of Gauze No. 1 agar colonies	White to grayish green, circular, dry, convex, undulate, non-smooth	NT*
Gram stain	G^+	NT
Form	Filamentous	NT
Spore chain morphology	Rectiflexibiles	Rectiflexibiles
Pigment production	-	-
Catalase	+	NT
Oxidase	+	NT
Nitrate reduction	+	+
Gelatin liquefaction	+	+
Starch hydrolysis	+	+
Casein hydrolysis	+	+
Cellulose hydrolysis	+	+
**Hydrogen sulfide production**	**+**	**-**
Growth between pH values	pH 5.0 to 10.0	NT
Growth between temperatures	28°C to 50°C	NT
**Growth in presence of NaCl**	**2% to 5%**	**< 4%**
Glucose	+	NT
L-Arabinose	-	-
L-Rhamnose	-	-
Sucrose	-	-
D-Xylose	-	-

D-Fructose	-	-
**Mannitol**	**-**	**+**
Raffinose	-	-
Inositol	-	-

NOTE: “+”Positive; “-” Negative; *NT, Not tested.

Supplementary Table 4 | Significantly differentially expressed protease genes in feather medium, compared with LB medium, cultured SCUT-3.

Gene ID	Pfam Annotation	MEROPS Family	Gene Name	Log_2 (FC)	FDR	Signal-P
FPDLIANG_04389	Subtilase family	S08A	*sep39*	8.88	7.15E-72	Y
FPDLIANG_01577	Thermolysin metallopeptidase, catalytic domain	M04	*sep69*	5.87	7.31E-23	Y
FPDLIANG_00055	NlpC/P60 family	C40	*cp17*	3.37	4.42E-29	Y
FPDLIANG_01876	Subtilase family	S08A	*sep53*	3.30	4.77E-13	Y
FPDLIANG_05098	Peptidase family M28	M28A	*sep33*	2.98	4.74E-05	Y
FPDLIANG_04554	Subtilase family	S08A	*sep40*	2.61	5.93E-04	Y
FPDLIANG_01877	Subtilase family	S08A	*sep43*	2.38	1.01E-05	Y
FPDLIANG_05369	Thermolysin metallopeptidase, catalytic domain	M04	*mp56*	2.33	2.29E-12	Y
FPDLIANG_00425	D-ala-D-ala dipeptidase	M15D	*mp32*	2.04	1.99E-07	Y
FPDLIANG_00108	Trypsin	S01E	*sep30*	1.93	1.21E-02	Y
FPDLIANG_01085	X-Pro dipeptidyl-peptidase (S15 family)	S15	*dpp58*	1.76	1.63E-03	Y
FPDLIANG_04544	Trypsin	S01E	*sep32*	1.68	2.36E-02	Y
FPDLIANG_03589	-	M06	*p47*	1.65	5.40E-04	Y
FPDLIANG_00948	Subtilase family	S08A	*sep44*	1.53	1.11E-07	Y
FPDLIANG_00832	Zinc carboxypeptidase	M14A	*mcp49*	1.52	8.59E-03	Y
FPDLIANG_05441	Streptomyces extracellular neutral proteinase (M7) family	M07	*mep19*	1.06	1.27E-02	Y
FPDLIANG_00666	Immune inhibitor A peptidase M6	M06	*p85*	1.05	2.94E-04	Y
FPDLIANG_00613	-	M23A	*mep45*	1.04	1.24E-02	Y
FPDLIANG_01422	Subtilase family	S08A	*sep46*	1.00	1.64E-04	Y
FPDLIANG_03721	Subtilase family	S08A	*sep113*	-1.22	2.66E-06	Y
FPDLIANG_00318	Trypsin	S01A	*sep59*	-1.23	6.13E-06	Y
FPDLIANG_01565	Beta-lactamase	S12	*scp46*	-1.86	1.14E-09	Y

FPDLIANG_03430	Putative modulator of DNA gyrase	M103	*mp48*	5.81	1.28E-37	N
FPDLIANG_03431	Putative modulator of DNA gyrase	M103	*mp47*	4.82	4.68E-26	N
FPDLIANG_00371	Metallopeptidase family M24	M24B	*ap54*	3.87	2.77E-16	N
FPDLIANG_00372	Metallopeptidase family M24	M24B	*ap52*	2.88	5.21E-08	N
FPDLIANG_01960	Peptidase family M48	M48B	*mep58*	2.85	1.33E-13	N
FPDLIANG_05339	Peptidase family M48	M48B	*mep76*	2.05	7.54E-11	N
FPDLIANG_00541	Peptidase family M48	M56	*mep34*	1.42	5.96E-05	N
FPDLIANG_01573	L,D-transpeptidase catalytic domain	C82A	*ptp43*	1.39	5.58E-06	N
FPDLIANG_04465	Peptidase family M23	M23B	*mp33*	1.24	1.14E-02	N
FPDLIANG_04319	Peptidase family M1 domain	M01	*ap82*	1.07	1.44E-02	N
FPDLIANG_02565	Sortase domain	C60A	*p27*	-1.01	3.22E-03	N
FPDLIANG_02946	Rhomboid family	S54	*sep19*	-1.07	2.40E-03	N
FPDLIANG_03975	Phosphoribosyl transferase	-	*cp92*	-1.10	1.26E-03	N
FPDLIANG_04257	Signal peptidase (SPase) II	A08	*aep23*	-1.15	1.09E-03	N
FPDLIANG_02937	DJ-1/PfpI family	C56	*p20*	-1.89	2.44E-08	N
FPDLIANG_04058	Beta-lactamase	S12	*scp39*	-1.89	2.11E-05	N
FPDLIANG_01311	Peptidase S24-like	S24	*sep34*	-1.92	3.79E-09	N
FPDLIANG_05223	Rhomboid family	S54	*sep29*	-2.26	1.06E-06	N
FPDLIANG_02266	Trypsin-like peptidase domain	S01B	*sep36*	-2.65	1.43E-12	N
FPDLIANG_03004	Peptidase family M48	M48B	*mep31*	-2.85	6.28E-15	N
FPDLIANG_05054	Peptidase family M48	M56	*mep33*	-4.38	2.49E-49	N

Supplementary Table 5 | Primers used in real-time PCR and PCR for gene expression verification.

Primer	Primer sequence (5′–3′)
16S rRNA F	ACGGGCAGGCTAGAGTTCGGT
16S rRNA R	GCTCCTCAGCGTCAGTATCGG
cdo-1 F	GCACCTGGTCGGCGAGAGA
cdo-1 R	CGAGGGAGACGAGGGAGAA
cdo-2 F	GGCGTGCTCACCGTGCTCC
cdo-2 R	TTCCAGCGAGTCGTTGACG
mdeA F	CCGAGCCCGACCCCAGCTA
mdeA R	AGGAGGAAGGCGGAGTGCG
tauE F	GCCTTCGGCCTGATGATGGTG
tauE R	TTCGGTGTGGACGTTCTTGGG
mshA F	TTCGGGCAGGTGCGTCAGG
mshA R	GGTGCAGCGGGGAGGTGTG
mshD F	CTCGTCCACACGGTGCTCG
mshD R	AAGTACTCGCCCCAGGCGG
sep39 F	GGCAACGACAACCAGG
sep39 R	TTGCTGAACGACGCCT
sep39 F	CGCCGAACAAGCTCCTCAA
sep39 R	GGTGCCCCTGTCCACGATG
sep53 F	GCCAACCACTTCTTCTACCT
sep53 R	GTCAGGGCCTTGTACCAGAT
m56 F	TTCTGGTCCGACTCCTGCTT
m56 R	CCGTGGGTCATCTCGTGGGCG
cp17 F	ACAACAAGACCCGCCACCTG

cp17 R	CTTCACCACCGAACCCGCCT
m32 F	CGCCGAACAAGCTCCTCAA
m32 R	GGTGCCCCTGTCCACGATG
sbnA F	CGTCTGTGTCACCGACCCCA
sbnA R	CTCACGGACGAGTGCCTTGC
catE F	GGCTGATGCTGACCCTGTGG
catE R	GCGCACCTCCTCGATGGAGT
yfiY F	AACAGGTCGTCGCCCAGTTG
yfiY R	CTTCTTGTTCAGCGCCTCCC
yfiZ F	TCTCAACGGGCGCGACTACG
yfiZ R	CCCAGCGAGAGGGCCACTTC
yusV F	CGAGCTGTCGGGCGGTCA
yusV R	GTCGAGTGCGCTGGTGGG
entS F	CGTCGCTGAGGAGGATCG
entS R	GGCCAGGAACGGGAAGAG
ArcB F	TGGACGGCATCAAGTGGA
ArcB R	CAGGCAGGCGTAGGGGTA
ndh F	CATCTTTAGCCGCAACCG
ndh R	CCTTCTCCGCGTACTGGG
dosT F	CGGCGGCGACTGGTACGA
dosT R	TGTTGCTGACCCGGCTCA
catD F	CCTTCTGGACCCTGTTCAT
catD R	CATGGTGGAGATGGTGTTG
Sep39-NdeI-F	GAGCGACATATGAAGCGTTTCCGGATCG CAGCCC
Sep39-EcorI-R	GAATTCGATATCTCAGAGGCCGGACTTG AAC
