# Supplementary material for: The feather degradation mechanisms of a new Streptomyces sp. isolate SCUT-3
Source: Commun Biol. 2020 Apr 24;3:191. doi: 10.1038/s42003-020-0918-0 (PMC7181669; doi:10.1038/s42003-020-0918-0)
Supplement: Supplementary file 2 — Description of Additional Supplementary Files [file 42003_2020_918_MOESM2_ESM.pdf]

Description of Additional Supplementary Files

File Name: **Supplementary Data 1**
Description: Source data used for graphs shown in Figure 1.

File Name: **Supplementary Data 2**
Description: Source data used for graphs shown in Figure 2.

File Name: **Supplementary Data 3**
Description: Source data used for graphs shown in Figure 3.

File Name: **Supplementary Data 4**
Description: Source data used for graphs shown in Figure 4.

File Name: **Supplementary Data 5**
Description: Source data used for graphs shown in Figure 5.

File Name: **Supplementary Data 6**
Description: Source data used for graphs shown in Table 1 and Supplementary Figures 1 and 7.
