# Supplementary material for: The feather degradation mechanisms of a new Streptomyces sp. isolate SCUT-3
Source: Commun Biol. 2020 Apr 24;3:191. doi: 10.1038/s42003-020-0918-0 (PMC7181669; doi:10.1038/s42003-020-0918-0)
Supplement: Supplementary file 3 — Reporting Summary [file 42003_2020_918_MOESM3_ESM.pdf]

nature research

Corresponding author(s): Xiao-Chun Luo

Last updated by author(s): Mar 26, 2020

Reporting Summary

Nature Research wishes to improve the reproducibility of the work that we publish. This form provides structure for consistency and transparency in reporting. For further information on Nature Research policies, see Authors & Referees and the Editorial Policy Checklist.

Statistics

For all statistical analyses, confirm that the following items are present in the figure legend, table legend, main text, or Methods section.

n/a	Confirmed	
☐	☒	The exact sample size (n) for each experimental group/condition, given as a discrete number and unit of measurement
☐	☒	A statement on whether measurements were taken from distinct samples or whether the same sample was measured repeatedly
☐	☒	The statistical test(s) used AND whether they are one- or two-sided<br>*Only common tests should be described solely by name; describe more complex techniques in the Methods section.*
☒	☐	A description of all covariates tested
☒	☐	A description of any assumptions or corrections, such as tests of normality and adjustment for multiple comparisons
☐	☒	A full description of the statistical parameters including central tendency (e.g. means) or other basic estimates (e.g. regression coefficient) AND variation (e.g. standard deviation) or associated estimates of uncertainty (e.g. confidence intervals)
☐	☒	For null hypothesis testing, the test statistic (e.g. F, t, r) with confidence intervals, effect sizes, degrees of freedom and P value noted<br>*Give P values as exact values whenever suitable.*
☒	☐	For Bayesian analysis, information on the choice of priors and Markov chain Monte Carlo settings
☐	☒	For hierarchical and complex designs, identification of the appropriate level for tests and full reporting of outcomes
☒	☐	Estimates of effect sizes (e.g. Cohen's d, Pearson's r), indicating how they were calculated

Our web collection on statistics for biologists contains articles on many of the points above.

Software and code

Policy information about availability of computer code

Data collection	SoftMax Pro 7.0<br>LightCycler96
Data analysis	Prokka, 1.12; BLAST+, 2.8.1; IntetProScan, 72.0; FastQC, 0.11.8; HISAT2, 2.1.0; StringTie, 1.3.5; SAMtools, 1.9; edgeR, 3.24.3; clusterProfiler, 3.10.1; IGV, 2.4.19; FGENESB, 24 Oct 2016; SignalP 4.1; gffutils, 0.8.4rc1; Biopython, 1.73; R, 3.5.2; Python, 3.6.3;GraphPad Prism 8.0

For manuscripts utilizing custom algorithms or software that are central to the research but not yet described in published literature, software must be made available to editors/reviewers. We strongly encourage code deposition in a community repository (e.g. GitHub). See the Nature Research guidelines for submitting code & software for further information.

Data

Policy information about availability of data

All manuscripts must include a data availability statement. This statement should provide the following information, where applicable:

- Accession codes, unique identifiers, or web links for publicly available datasets
- A list of figures that have associated raw data
- A description of any restrictions on data availability

The genome of SCUT-3 (Gene Bank accession number: CP046907)
The RNA-seq data used in this study are deposited in the National Center for Biotechnology Information SRA database (SRA accession no. PRJNA611875).
The 16s rRNA of SCUT-3 (Gene Bank accession number MK743936.1)
The source data underlying the graphs and charts presented in the main figures are shown as Supplementary Data 1-5.
All data generated or analysed during this study are included in this published article (and its supplementary information files).

Field-specific reporting

Please select the one below that is the best fit for your research. If you are not sure, read the appropriate sections before making your selection.

☒ Life sciences ☐ Behavioural & social sciences ☐ Ecological, evolutionary & environmental sciences

For a reference copy of the document with all sections, see nature.com/documents/nr-reporting-summary-flat.pdf

Life sciences study design

All studies must disclose on these points even when the disclosure is negative.

Sample size	Sample size was indicated in the figure legends and source data of each experiment.
Data exclusions	No data was excluded from analyses.
Replication	All experiments were independently repeated in triplicates.
Randomization	For animal studies, Rice seeds and 10 gram tilapias were assigned randomly to the experimental and control groups.
Blinding	Investigators were not blinded.

Reporting for specific materials, systems and methods

We require information from authors about some types of materials, experimental systems and methods used in many studies. Here, indicate whether each material, system or method listed is relevant to your study. If you are not sure if a list item applies to your research, read the appropriate section before selecting a response.

Materials & experimental systems

n/a	Involved in the study	
☒	☐	Antibodies
☒	☐	Eukaryotic cell lines
☒	☐	Palaeontology
☐	☒	Animals and other organisms
☒	☐	Human research participants
☒	☐	Clinical data

Methods

n/a	Involved in the study	
☒	☐	ChIP-seq
☒	☐	Flow cytometry
☒	☐	MRI-based neuroimaging

Animals and other organisms

Policy information about studies involving animals; ARRIVE guidelines recommended for reporting animal research

Laboratory animals	Nile Tilapia (Oreochromis niloticus) was purchased from Guangdong Tilapia Fine Germchit Field (Guangdong, China). About 7 gram Nile Tilapia were used in this manuscript.
Wild animals	No wild animals involved in this article manuscript.
Field-collected samples	This manuscript does not involve collected samples from field.
Ethics oversight	Tilapia nutrition experiment was performed in the Aquaculture Laboratory of College of Marine Science, South China Agricultural University, and was approved by the Experimental Animal Ethics Committee of South China Agricultural University.

Note that full information on the approval of the study protocol must also be provided in the manuscript.
